# Cardiovascular Nursing Workforce Challenges: Transforming the Model of Care for the Future

**DOI:** 10.14797/mdcvj.1188

**Published:** 2023-03-07

**Authors:** Gail M. Vozzella, Michelle C. Hehman

**Affiliations:** 1 Houston Methodist Hospital, Houston, Texas, US; 2 Houston Methodist Academic Institute, Houston, Texas, US

**Keywords:** cardiovascular nursing, nurse workforce, nurse shortage, work environment, model of care

## Abstract

The complexities of acute and critical care cardiovascular management demand specialty trained and experienced nurses to ensure quality patient outcomes. An ongoing nurse labor shortage threatens to destabilize the healthcare system and presents a twofold challenge: a decreasing supply of registered nurses and increasing demand for nursing services. This article describes the numerous forces driving the current nursing shortage as well as the impact of the coronavirus-19 pandemic on nurse job satisfaction and turnover. We present a reinvented model of nursing care as a framework for healthcare organizations to address nurse staffing challenges.

## Introduction

High-quality cardiovascular (CV) nursing requires a specialized mix of cardiac scientific knowledge, astute assessment skills in changing patient conditions, and the ability to react quickly with appropriate nursing interventions. In recent years, cardiac nursing has become progressively more complex, driven largely by advances in medical science such as supportive technology, medication, and device therapies as both a bridge to transplantation or destination therapy. These innovative tools, as well as expanded surgical and interventional techniques, have broadened competency requirements for CV nurses. Higher-acuity patients also increase nursing workload demands, a trend that will likely continue amid rising CV disease burden and a higher prevalence of comorbid conditions among hospitalized patients.^1,2^ A significant body of literature exists to demonstrate the impact of nursing workload, skill mix, and staffing on the quality of patient care.^3,4,5,6,7^ Having specialty trained and certified CV nurses has been shown to help decrease delays in cardiac referral and treatment, reduce hospital stays in patients with cardiac disease, and prevent rehospitalization.^8,9,10^ Against the backdrop of an ongoing nurse shortage, healthcare organizations need strategic plans to support, build, and retain a strong team of CV nurses.

As the largest hospital workforce, nurses play a critical role in the stability of the healthcare system. The coronavirus-19 (COVID-19) pandemic underscored the gravity of acute nurse shortages, bringing renewed attention to the importance of a strong healthcare workforce. Since the early 1900s, the United States (US) has faced periodic nurse shortages as particular sociopolitical, economic, or technological forces rapidly escalate the demand for nursing labor.^11,12^ The current nurse shortage, however, also involves a decreasing supply of registered nurses (RNs) at the same time the demand for services continues to increase, leading to an ever-widening nurse labor deficit.^13,14^ Coordinating a lasting and effective response will require a nuanced understanding of the complex factors involved. This article reviews the numerous forces driving the shortage to identify areas of opportunity for CV nursing leadership to address staffing challenges. We also describe a new model of nursing care, providing a framework for healthcare organizations to improve the work environment by reducing workload burden and enhancing educational support for clinical staff.

## The Nurse Labor Shortage

Nursing leaders and global health organizations have long warned about the growing nurse labor shortage as the world’s population lives longer and requires more healthcare services.^15^ In the US, researchers estimate that due to the rising prevalence of chronic illness and an aging baby boomer generation, hospital utilization will increase 40% by the year 2025.^16,17^ Future growth of the nursing workforce, expected to increase 6% over the next decade, will not keep pace with the rising need for nursing services.^18,19^

For more than a decade, predictive workforce models have anticipated a nurse staffing crisis as demand continues to exceed supply; recent projections have become more worrisome as forecasts incorporate the additional impact of the COVID-19 pandemic on nurse retention.^14^ While nurse labor shortages impact all practice areas, the highly specialized nature of modern CV care requires adequate numbers of appropriately skilled and experienced nurses to ensure quality and safety. Even temporary staffing disruptions cause challenges for experienced CV nurses, who express frustration and worry when working with an inexperienced or constantly changing nursing team.^20^ Nurse staffing issues did not begin with the pandemic, but the events of the last 3 years have placed immeasurable strain on the entire healthcare system, exacerbating existing issues and exposing latent weaknesses. Due to the multifaceted nature of the nurse shortage, healthcare organizations are challenged to understand how all these factors influence staffing.

## Nursing Workforce Issues

The US Bureau of Labor Statistics estimates the current RN workforce at 3,047,530 nurses.^18^ Cardiovascular nursing is the eighth largest inpatient specialty, with nearly 3% of nurses specializing in cardiac or CV care.^21^ Recent demographic surveys reveal shifts in race and ethnicity, educational attainment, and age distribution of nurses. While the profession is still predominantly white and female, younger nurses have increased the overall racial and ethnic diversity of the profession, making it more representative of the US population than ever before.^18^ This trend holds promising potential for reducing health disparities and improving equity and access to healthcare services because racial and language concordance between patients and providers improves communication, trust, and decision-making.^22,23^ The RN workforce also is becoming more educated and specialized, both of which correlate with improved care quality.^4,24^ Nearly half of all RNs report a bachelor’s degree as their highest level of nursing education, and more than 1 million hold one or more nursing specialty credentials.^18,25^

**Figure 1:**
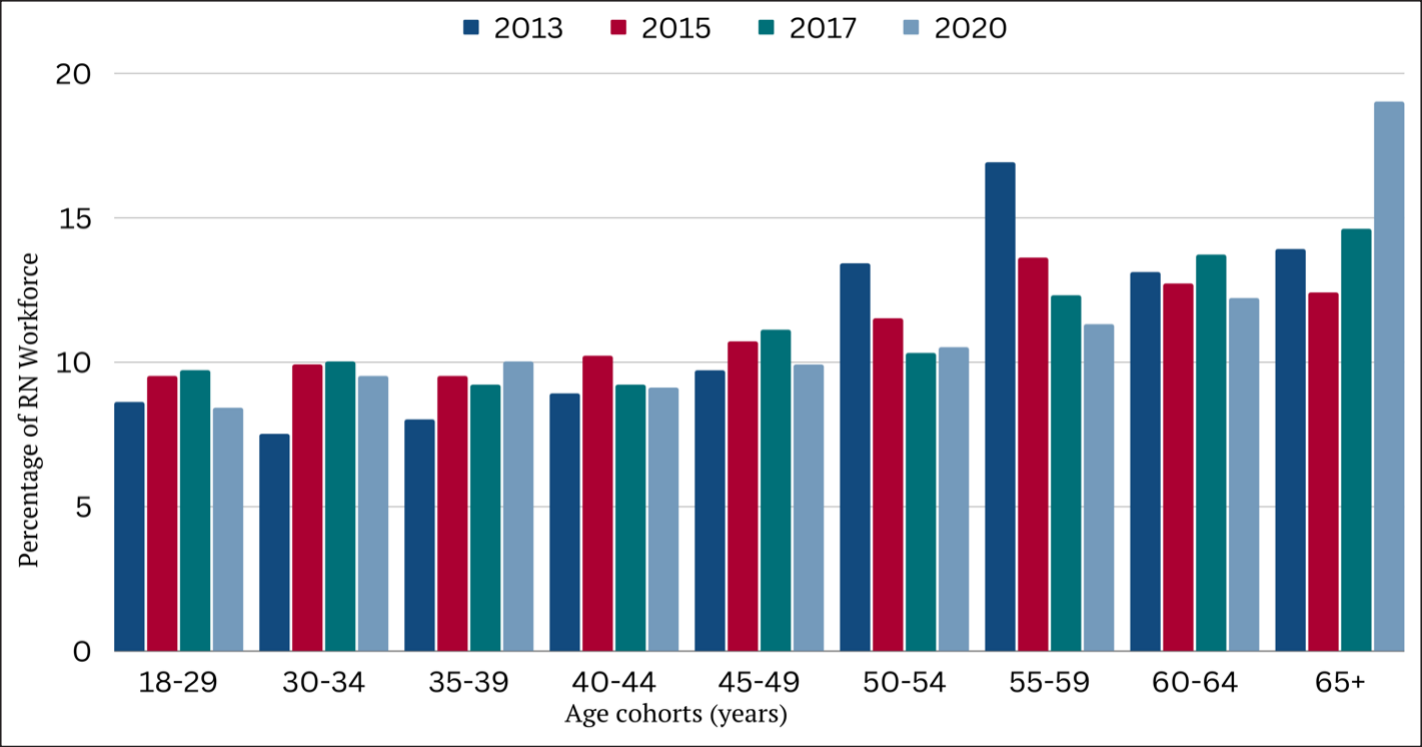
Age distribution of Registered Nurses (RNs) by year. The median age of RNs is 52 years and has remained consistent over the last decade. However, the overall age distribution of the nursing workforce has shifted significantly since 2013, with more than half of all RNs over the age of 50.^13^

Overall, the nursing profession is aging. In 2020, the median age of RNs rose to 52 years amid substantial shifts in age distribution across the profession (Figure 1).^13^ Nurses aged 65 years or older now comprise the largest single age cohort, with 19% of the workforce already at typical retirement age. More alarming, an even greater proportion of the total nurse workforce is *nearing* retirement, with 47% of nurses over the age of 50.^13,26^ Nearly half the workforce could retire in the next 10 to 15 years, a striking reduction both in sheer numbers and professional expertise. Collectively, older nurses possess an extensive amount of clinical proficiency, expert knowledge, and mentorship ability, and researchers anticipate an annual loss of more than 2 million years of nursing experience as these nurses retire.^27^

The implications of an aging nurse workforce extend beyond the clinical environment. Just as a significant percentage of clinical RNs near retirement, an even greater proportion of nurse faculty members are nearing retirement age, threatening to further upend an already strained nurse education system.^28^ Future growth of the profession depends upon a robust educational pipeline to ensure an adequate number of new nurses join the workforce each year. Despite substantial interest in the profession, nurse training programs lack sufficient capacity and are forced to turn away tens of thousands of qualified candidates each year. In 2021 alone, entry-level baccalaureate programs rejected 76,140 applications due to facility constraints, a lack of clinical placements, and/or a shortage of nursing faculty.^29^ With more than 63% of full-time nursing faculty members aged 50 or older, the nurse educator shortage will continue to worsen, further reducing educational capacity and restricting the growth potential of the profession.^13^

While the looming threat of an aging RN workforce has long been an issue of concern, the impact of COVID-19 on nurse turnover threatens to further destabilize the system. Nurse turnover is defined as any job move that results in either an internal shift between roles or an external move out of an organization or the profession entirely.^30^ High turnover rates negatively impact the quality of patient care, increase the financial and time resource burden of the healthcare system, and exacerbate nurse labor shortages.^31,32,33,34^ Disruptions in the healthcare delivery system throughout the COVID-19 pandemic predictably raised turnover rates as nurses adapted to changing work patterns, dealt with elder care and child care closures, and sought opportunities for travel nursing or remote telehealth positions.^33^ As a result of these challenges, the size of the nursing workforce initially plateaued, then dropped significantly when more than 100,000 RNs left the workforce in 2021, the largest single-year drop in 40 years.^27^

**Figure 2:**
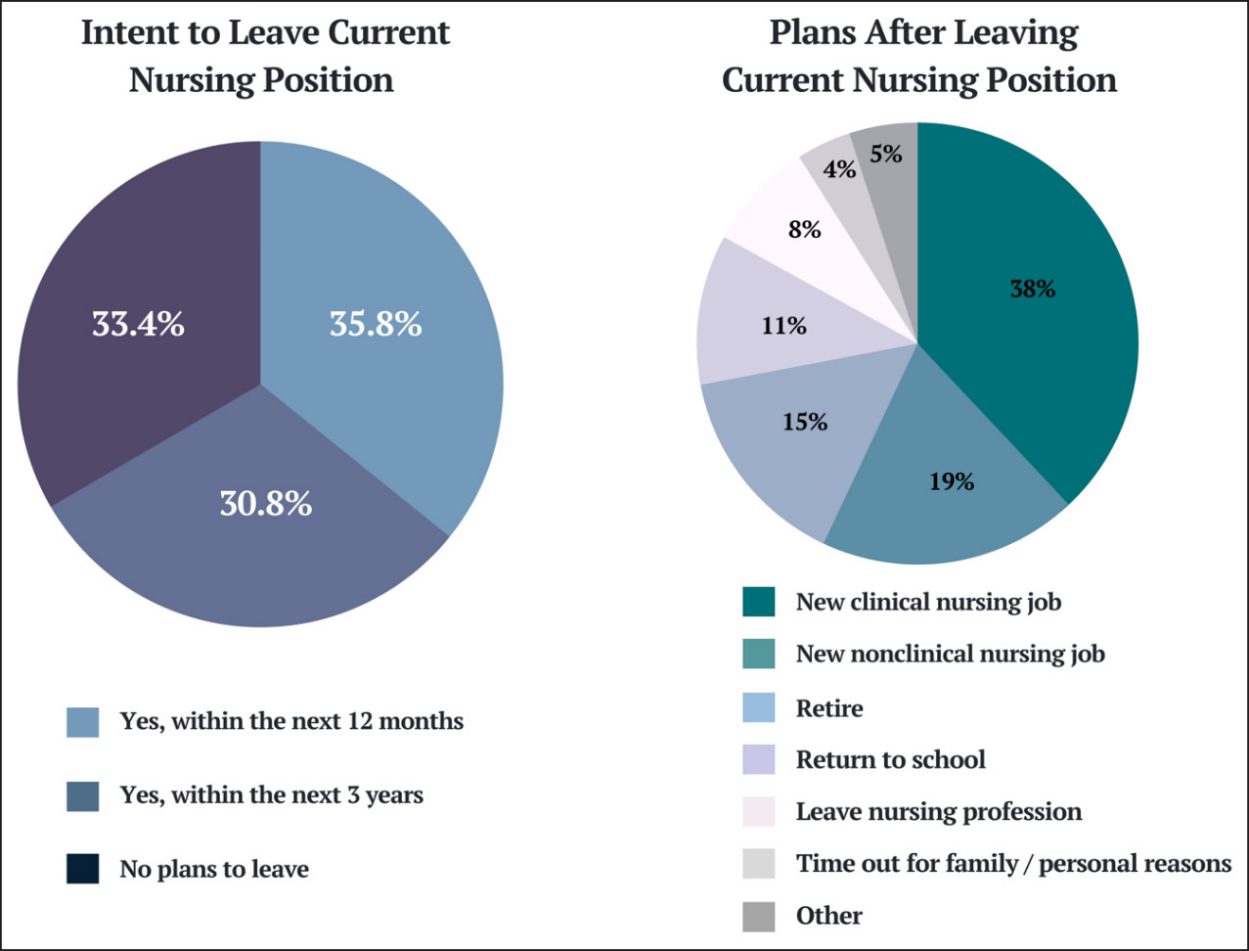
Intention to leave nursing and plans of those who intend to leave their position. A 2021 national survey of nurse work environments found that two-thirds of nurse respondents planned to leave their current position within the next 3 years.^33^ Of the respondents with plans to leave, only 38% intended to find a new position as a clinical nurse.

Measuring and anticipating nurse turnover is difficult because no standard operational definition currently exists. Instead, researchers evaluate individual intention to leave as the most accurate predictor of future nurse turnover, and recent data indicate that turnover intention has continued to rise. Between 22% and 44% of RNs intend to leave their current position within the next year, and 67% plan to leave within the next 3 years (Figure 2).^31,32,33,35^ Of the nurses who express intent to leave, only 38% plan to find a different position in clinical nursing, with most respondents planning to seek nonclinical opportunities or leave the profession entirely.^33^ This type of turnover, where nurses leave direct caregiving roles, significantly exacerbates the labor shortage, particularly when seen among early career nurses. This trend is likely to continue as nurses aged 35 and younger report the highest turnover intention as well as the lowest rates of job satisfaction and emotional well-being.^35^ Nurse emotional health, job satisfaction, and burnout are all correlated with key elements of the work environment.^31,33^

## Work Environment Issues

Practicing in a workplace that is safe, empowering, and satisfying allows nurses to provide the highest quality care to patients and families while also fostering personal and professional development. A growing body of scientific literature demonstrates the influence of workplace health on quality indicators and patient outcomes.^36,37^ Healthier work environments are correlated with higher survival rates following cardiac arrest, lower 30-day inpatient mortality rates, lower rates of failure-to-rescue, and a reduction in factors associated with missed care.^3,7,38,39^ Nurses who practice in healthier workplaces report better overall psychological health, record greater job satisfaction, and demonstrate higher work engagement.^37,40^ A healthy work environment also is correlated with a lower incidence of workplace injury and lower nurse turnover intention.^33,41,42,43,44^ Recognizing these implications, the World Health Organization, National Academy of Medicine, and American Association of Critical Care Nurses have all emphasized the importance of promoting workplace health.^34,45,46^

The COVID-19 pandemic brought unprecedented challenges to the healthcare work environment. Frontline caregivers dealt with acute staffing shortages related to surging patient volume and acuity, continuously changing practice protocols, unavoidable exposure risks, crisis standards of care, inequities in disease risk and burden among already marginalized communities, and the collective trauma of millions of lives lost, many of whom were healthcare providers. Though the intensity of the crisis has waned, the emotional toll of delivering care during a global disaster, coupled with sustained staffing shortages, higher acuity patients, and increasing workload demands, leaves healthcare providers feeling unsupported in their work environment.^34,47^

Critical care nurses, particularly those who work in cardiac and CV intensive care units (ICUs), experienced high levels of burnout and increased turnover even before the pandemic began, attributed to the physical, emotional, and intellectual demands of their practice environment.^48,49,50,51,52^ COVID-19 intensified these workplace stressors, and CV nurses may have shouldered an even greater burden. Their specialized knowledge and expert clinical judgment proved invaluable during the pandemic given the high incidence of CV complications with COVID-19, but these skills also meant an increased likelihood of caring for the most complex and clinically demanding patients.^20^ As a result, ratings of the current nurse work environment are lower than ever before, driven largely by burnout, a lack of organizational resources, and unrealistic workload assignments.^33,48,49,50,51,51^

Individual nurse workloads are the combined result of staffing ratios, nursing skill mix, patient acuity, hospital throughput, technology management demands, and the availability of support personnel. High workload stress occurs when nurses are expected to complete too many tasks in the available amount of time. Nursing staff who feel unprepared for practice, either from a lack of experience or educational support, also can perceive higher workload burdens and experience greater work-related stress.^53,54,55^ Today’s hospitalized CV patient requires more complex medical management and sophisticated nursing care even as hospital operations have begun to focus more on throughput and cost containment.^56^ Nursing responsibilities have expanded and patient acuity has increased—all while support personnel teams have been reduced and nurse staffing ratios have worsened. The resulting increase in workload burden is correlated with lower job satisfaction and higher intent to leave among direct care nurses.^57,58^

**Figure 3:**
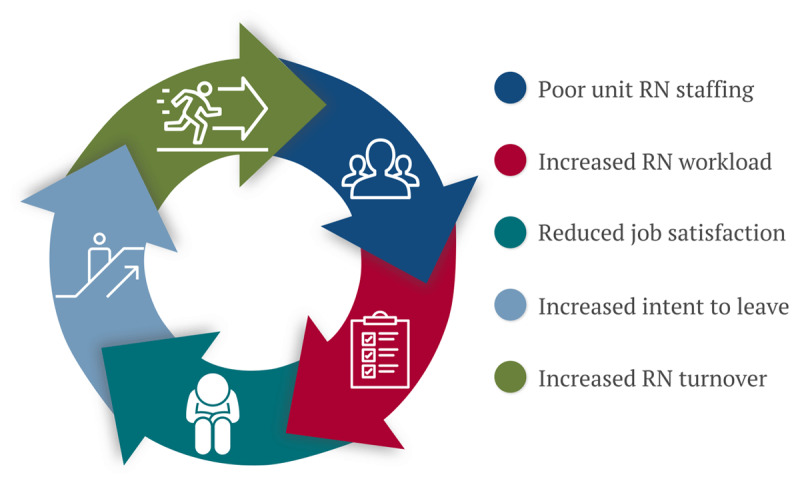
Relationship between staffing, workload stress, job satisfaction, and turnover. Poor registered nurse (RN) staffing on clinical units leads to increased individual nursing workloads. Over time, this can reduce overall job satisfaction and increase an individual RN’s intention to leave their current position. Intent to leave is strongly correlated with future nurse turnover and, as attrition rates climb, unit staffing worsens and perpetuates the whole cycle.

Nurse workforce characteristics and work environment issues are deeply interconnected, and no single force works in isolation to drive the current nurse shortage. For example, both individual and environmental factors shape nurse job satisfaction, which in turn influences turnover intention and eventual attrition from the job or the profession. In times of critical RN shortages, poor staffing leads to increased workload demands on the remaining RNs and contributes to low job satisfaction. Over time, poor job satisfaction fuels intent to leave and increases turnover, which worsens staffing on the unit and creates additional patient care workload burdens. These issues magnify and perpetuate one another, creating a continuous negative cycle between staffing, workload, job satisfaction, and turnover (Figure 3). To address the myriad challenges presented by the current nurse shortage, organizations must look toward comprehensive, systems-level strategies to improve retention by adding additional workplace support.

## Shaping a New Model of Nursing Care

The current nurse shortage presents an opportunity to revolutionize traditional paradigms for nursing education and practice. Nursing leadership and healthcare organizations must focus on developing innovative plans with sustainable impact that address the immediate needs of nurse clinicians. The reinvented model of nursing care at Houston Methodist (HM) prioritizes the needs and experiences of clinical RNs while also acknowledging the reality of a worsening nurse shortage and the likelihood of continued staffing challenges. Given the magnitude and forecast of the current staffing crisis, primary and team-based models of nursing no longer offer a feasible template for providing safe and effective patient care. Rather than asking nurses to do more, the HM model of care integrates creative approaches to technology with enhanced education and training support to ensure high-quality, exceptional patient care regardless of nursing workforce transitions. The HM nursing model incorporates virtual ICU nursing, acute care telenursing, and an expanded nursing education team into the traditional nursing practice model, providing bedside clinicians with the tools and structural support necessary to work safely and efficiently at the highest level of their licensure. By reducing task-based workload, utilizing virtual nursing to supplement bedside clinicians, and expanding the number of unit-based nurse educators, the new care model maximizes the experience, skill mix, and size of the nursing workforce by improving efficiency without sacrificing care quality.

## Leveraging Digital Technology for Nursing Practice

New models of nursing must streamline care delivery processes and enhance communication among patients and providers.^15,34,35,59^ By leveraging the pandemic-driven expansion of virtual nursing services into acute and intensive care settings, technology becomes a nurse extender, capable of improving patient safety while offloading tasks from bedside caregivers. This hybrid model of care integrates digital technology into the nursing workflow and provides real-time support to bedside clinicians from equivalently skilled and licensed RNs. At HM, a remote nursing operations center houses both a virtual intensive care unit (vICU) and a virtual acute care telenursing program that provide direct support to clinical RNs on dedicated patient care units across the hospital.

In the vICU, experienced critical care nurses located in the remote operations center partner with bedside intensive care teams to provide enhanced observation of critically ill patients, enabling earlier identification of potential decompensation. Virtual coverage extends to four separate ICUs, including the cardiac ICU and CVICU. A total of 126 ICU beds are monitored by vICU nursing staff. High-resolution cameras and two-way audiovisual equipment allow for continuous, unobtrusive patient observation and remote consults at the bedside. The remote nursing team is composed of experienced cardiac critical care nurses who use their specialized knowledge, keen observation skills, and advanced critical thinking ability to integrate real-time physiological data with patient-specific risk factors. Combined with predictive analytic tools, the enhanced monitoring and observation allows the virtual nursing team to anticipate status changes and initiate early interventions.

A separate virtual nursing unit was recently initiated to assist with admission and discharge procedures on acute care floors. The acute care telenursing staff provide virtual support to four separate inpatient units with a total of 137 beds across the hospital. Coverage includes units managing cardiac and CV patients, which frequently have complex medical histories, medication lists, and treatment modalities that make the admission and discharge process more time-consuming for nurses. Bedside RNs on these units also have higher nurse-to-patient ratios, increasing the potential for interruptions during these vital beginning and ending moments of an inpatient stay. When a clinical nurse requests support from the telenursing team, patients are connected to an experienced virtual nurse via video conferencing software on a bedside smart tablet. The one-on-one interaction allows for a more focused and comprehensive process whereby nurses have more time to answer questions, provide in-depth teaching, and address any issues with care coordination. Favorable patient responses and positive reception by both the bedside and virtual RN teams have pushed plans for expansion of telenursing to additional hospital units and to broaden services to further reduce task-based workload burden and offer timely support to clinical nurses. Additional telenursing responsibilities may include double-checking high-risk medications or treatments, monitoring and coaching clinical RNs through procedures, or serving as an on-call resource for new staff.

This expanded hybrid model of nursing care aligns with recent evidence that indicates nurses are looking for technology integration that makes their work more efficient and flexible work options that maintain a connection to patient care.^33,35,60^ Defining and growing these new nursing roles meant that HM could offer an innovative work alternative to experienced RNs seeking a less physically demanding nursing position. These opportunities allowed the organization to remain competitive in a challenging RN job market while also improving retention of highly skilled CV nurses and protecting current staffing levels across the system.

## Redefining the Role of the Nurse Educator

Another key component of the new HM model of care involves the expansion of the nursing education department to allow for the placement of a dedicated Professional Practice Leader (PPL) on each patient care unit. The PPL role extends the educational support given to nurses during new employee orientation and the nurse residency program, providing continuous mentorship, practice oversight, and just-in-time education to clinical RNs throughout their career. Nurses who function in the PPL role are highly educated expert nurse clinicians responsible for developing individualized, specialty-specific training and professional development plans for direct care nurses on their unit. PPLs oversee the transition to independent practice by new nurses, serve as a resource for organizational policies and standards of care, and implement practice changes according to the latest scientific evidence. They also ensure unit outcomes meet quality standards through practice audits, and their work has already improved the completion, accuracy, and timeliness of physical assessment documentation among nursing staff.^61^

Throughout the pandemic, the existing PPL team expertly navigated continuous policy and procedure changes, the rapid evolution of scientific knowledge, and social distancing challenges during onboarding. PPLs also were instrumental in the rapid development of an organization-wide nurse labor pool to respond to COVID-19 surges, further highlighting the value of a robust nurse education department and the need to increase the team to boost unit-based support.

Enhancing PPL reach to every unit also may improve retention among novice and early career nurses by reducing transition-related stress.^53^ Transition stress, which results from a lack of mentorship and poor preparation for the complex demands of modern nursing practice, frequently leads to burnout and increased turnover.^54^ The complexities of modern CV nursing care increase the odds of transition stress. Nearly half of new nurses leave their position within the first year, primarily due to the large competency gap between educational preparation and professional practice; the added pandemic-related disruption of recent nurse graduate training experiences likely adds to stress and feelings of inadequacy.^53,54,55^ While a comprehensive nurse residency program like the one at HM has been shown to increase job satisfaction in novice nurses, the addition of a PPL on each unit provides a continuation of the residency’s supportive educational structure and allows for more individualized training strategies in the event of knowledge gaps or process changes. PPLs in the CICU, CVICU, and acute care cardiac units ensure that direct care nurses develop confidence in their professional practice through appropriate experiential learning opportunities.

## Looking to the Future

Future avenues for change to the HM model of care must be evidence-based and align with the strategic goal of reducing nurse workload burden while providing additional support for clinical staff. The relationship between nurses and technology is being restructured, and RNs are involved in every step of the technology integration process to ensure that new devices enhance nursing workflow. In addition to extending virtual nursing capabilities, pilot programs are being developed for smart devices that enhance physiologic monitoring, artificial intelligence-enabled predictive modalities, virtual reality skills training, and helper robots. Nurse researchers at HM also are initiating studies to evaluate the work environment across the organization to identify more specific areas of opportunity and tailor interventions according to nurse responses. Finally, nursing leaders are looking to enhance interprofessional communication and collaboration, which have been shown to independently improve nurse job satisfaction, reduce intention to leave, and improve patient outcomes.^62,63,64,65^

## Conclusion

The current nurse labor shortage presents healthcare organizations with unique challenges but also provides an ideal opportunity to revolutionize traditional models of care. At HM, innovative approaches to technology integration and educational support give nurses the resources they need to thrive in the current healthcare environment. The success of the HM model lies in its collaborative development process, solid technological infrastructure, and clearly defined responsibilities of the virtual care team and unit-based PPLs. Healthcare organizations will ultimately need to tailor approaches to their specific needs, but all strategic plans should include efforts that recognize the value of experienced nurses and invest in programs that support current staff and facilitate competency for novice nurses.

## Key Points

- The complex nature of modern cardiovascular management requires experienced, specialty trained nurses to ensure high-quality patient outcomes.
- The current nursing shortage presents a two-fold challenge as the supply of nurses continues to decrease at the same time demand for nursing services is on the rise.
- Major factors contributing to the nursing shortage include an aging RN workforce, higher rates of nurse turnover, and a more demanding healthcare work environment.
- A new model of nursing care at Houston Methodist incorporates virtual intensive care nursing, acute care telenursing, and an expanded nursing education team to provide bedside clinicians with the tools and structural support necessary to work safely and efficiently despite staffing challenges.
